# Enhancement of forward suppression begins in the ventral cochlear nucleus

**DOI:** 10.1016/j.brainres.2016.02.043

**Published:** 2016-05-15

**Authors:** Neil J. Ingham, Naoya Itatani, Stefan Bleeck, Ian M. Winter

**Affiliations:** Centre for the Neural Basis of Hearing, Department of Physiology, Development and Neuroscience, University of Cambridge, Downing Street, Cambridge, CB2 3EG, United Kingdom

**Keywords:** AC, auditory cortex, AN, auditory nerve, BF, best frequency, CF, characteristic frequency, Ch, chopper, CN, cochlear nucleus, CS, chopper-sustained, CT, chopper-transient, CV, coefficient of variation, DCN, dorsal cochlear nucleus, GoS, growth of suppression, IC, inferior colliculus, ICc, central nucleus of the inferior colliculus, ISI, inter-spike interval, ON, onset, OL, onset-L, OI, onset-I, OC, onset-chopper, OS, onset- sustained, P, pauser, PEST, parameter estimation by sequential testing, PL, primary-like, PN, primary-like with notch, PSTH, peri-stimulus time histogram, S, sustained, VCN, ventral cochlear nucleus, Guinea pig, Auditory, Brainstem, Masking, Context

## Abstract

A neuron׳s response to a sound can be suppressed by the presentation of a preceding sound. It has been suggested that this suppression is a direct correlate of the psychophysical phenomenon of forward masking, however, forward suppression, as measured in the responses of the auditory nerve, was insufficient to account for behavioural performance. In contrast the neural suppression seen in the inferior colliculus and auditory cortex was much closer to psychophysical performance. In anaesthetised guinea-pigs, using a physiological two-interval forced-choice threshold tracking algorithm to estimate suppressed (masked) thresholds, we examine whether the enhancement of suppression can occur at an earlier stage of the auditory pathway, the ventral cochlear nucleus (VCN). We also compare these responses with the responses from the central nucleus of the inferior colliculus (ICc) using the same preparation. In both nuclei, onset-type neurons showed the greatest amounts of suppression (16.9–33.5 dB) and, in the VCN, these recovered with the fastest time constants (14.1–19.9 ms). Neurons with sustained discharge demonstrated reduced masking (8.9–12.1 dB) and recovery time constants of 27.2–55.6 ms. In the VCN the decrease in growth of suppression with increasing suppressor level was largest for chopper units and smallest for onset-type units. The threshold elevations recorded for most unit types are insufficient to account for the magnitude of forward masking as measured behaviourally, however, onset responders, in both the cochlear nucleus and inferior colliculus demonstrate a wide dynamic range of suppression, similar to that observed in human psychophysics.

## Highlights

• A two-interval forced choice procedure was used to measure suppressed thresholds.
• The time course of recovery from suppression was fastest for onset units.
• Growth of suppression with increased suppressor level was greatest for onset units.
• Suppression in onset units was greater than that seen in auditory nerve fibres.

## 1 Introduction

In the natural auditory environment, single tones rarely occur in isolation; most often auditory signals are comprised of complex patterns of spectral and temporal information, against background ambient noise. The perception of salient signals is highly context dependent and our knowledge of the mechanisms of how such processing occurs is incomplete. One important aspect for processing auditory signals is masking, defined as “the process by which the threshold for one sound is raised by the presence of another (masking) sound” (Moore, 2003). Physiological correlates of psychophysical forward masking have been measured using a two-tone paradigm. The first tone (*aka* the masker) is assumed to suppress the response to the second tone (*aka* the probe). At the level of the auditory nerve this phenomenon has been attributed to peripheral short-term adaptation in response to the masker tone (Kiang et al., 1965, Harris and Dallos, 1979). The early results of forward masking studies in the AN (e.g. Harris and Dallos, 1979) showed some similarities with psychophysical data (e.g. the periods of recovery from masking have similar time courses), however, differences in other parameters suggest that central auditory pathways contribute to the psychophysical phenomenon of forward masking.

Auditory nerve fibres terminate in the cochlear nucleus (CN), and several studies have shown a hierarchy of forward suppressed responses from the different unit types in the CN (Palombi et al., 1994, Backoff et al., 1997, Shore, 1998, Boettcher et al., 1990, Bleeck et al., 2006). The range of forward suppressed responses found in the CN is likely to be a reflection of the synaptic arrangements, cell-membrane properties, and the interneuronal circuitry associated with each cell type. The studies in the CN (and some in the AN) considered forward suppression as the difference in number of spikes evoked by a probe stimulus in the presence and absence of a preceding conditioner tone (Harris and Dallos, 1979, Boettcher et al., 1990, Palombi et al., 1994, Shore, 1995, Bleeck et al., 2006). This approach is problematic as it is unknown how spike count ratios relate to a potential change in neural threshold. To overcome this problem several studies have now measured the neurometric thresholds of single neurons at the level of the IC (Nelson et al., 2009) and auditory cortex (AC; Alves-Pinto et al., 2010; Scholes et al., 2011). It has been argued that the amount of suppression found in unit populations in the inferior colliculus (IC) of the marmoset (Nelson et al., 2009) and the auditory cortex of the guinea pig more closely resembles the magnitude and time course of forward masking as observed psychophysically in humans. In the IC a simple neural circuit was hypothesised to account for the data although it is still unknown whether the amount of suppression observed in the IC is created de novo or is simply a reflection of processing carried out at an earlier stage in the processing pathway. To test this we have quantified the magnitude of forward suppression in terms of dB threshold changes of suppressed probe tones, using identical stimulus paradigms, for neurons in the VCN, and in the IC. Units classified as onset-like, in both the VCN and IC, showed suppression that was greater in magnitude than that observed in a similar study in the auditory nerve.

## 2 Results

### 2.1 Single unit classification

**Fig. 1:**
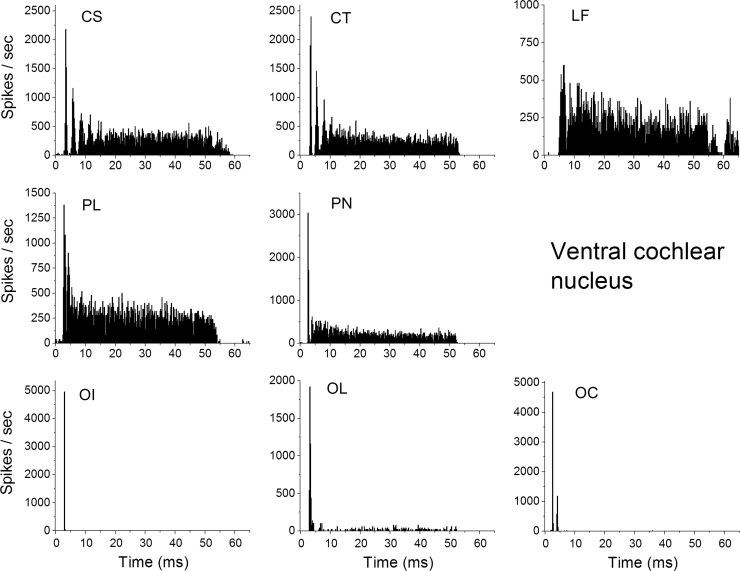
Examples of PSTH responses from the different unit types in the Ventral Cochlear Nucleus. Responses shown were recorded in response to 250 presentations of a 50 dB sensation level, random starting phase, 50 ms duration, best frequency (BF) tone. Neuron types illustrated are sustained chopper (CS), transient chopper (CT), low frequency (LF), primary-like (PL), primary-like-with-notch (PN), onset-I (OI), onset-L (OL) and onset chopper (OC).

Single unit recordings were obtained from 113 neurons in the left ventral CN of 27 animals. Neurons were classified according to their temporal discharge patterns in response to these BF tone bursts, using the schemes of Blackburn and Sachs (1989), Young et al. (1988) and Winter and Palmer (1995). This takes into account PSTH shape, distribution of inter-spike interval (ISI) and the coefficient of variation (CV) of discharge regularity (calculated by an average of the ratio of mean ISI, *µ*, and its standard deviation, *σ*, between 12–20 ms after onset). Examples of PSTHs for each class of neuron, recorded at 50 dB SL, are shown in Fig. 1. Primary-Like (PL) neurons had an exponential decline in ISI distribution. Primary-Like with Notch (PN) demonstrated a similar ISI pattern but had a pronounced reduction in PSTH spike rate subsequent to a precisely-timed first spike. Onset neurons had a precisely timed onset spike with a low standard deviation in first spike latency. Onset chopper (OC) neurons demonstrated a second peak in the PSTH response, especially at 50 dB above threshold, often with a low level sustained rate of activity. Onset-L (OL) neurons showed a similar response, but without the second peak. Onset-I (OI) neurons fired a single onset spike and showed no sustained rate even at high stimulus levels. Chopper units showed regular peaks in their PSTH; those with a CV value of less than or equal to 0.3 were classified as sustained choppers (CS), whereas those with a CV above 0.3 were defined as transient choppers (CT). Neurons with BFs below 0.5 kHz were routinely classified as low frequency (LF). Neurons that could not be classified by the above criteria were deemed unusual (UN) and are not discussed further. Using these criteria, the following distribution of neurons was recorded: OC (*n*=12), OI (*n*=6), OL (*n*=5), CS (*n*=18), CT (*n*=39), PL (*n*=9), PN (*n*=12), LF (*n*=10), UN (*n*=2).

**Fig. 2:**
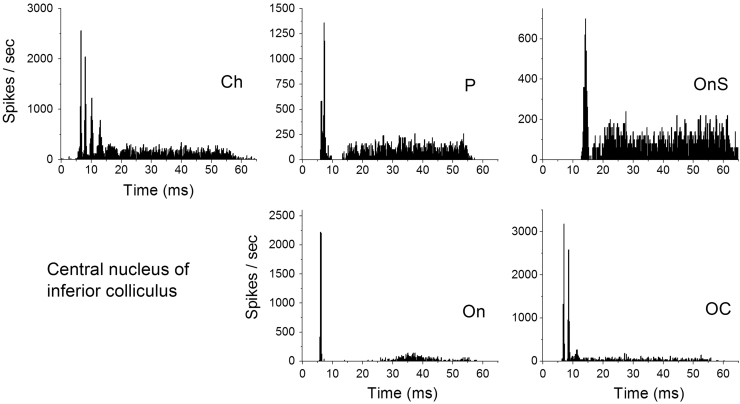
Examples of PSTH responses from the different unit types in the central nucleus of the Inferior Colliculus. Responses shown were recorded in response to 250 presentations of a 50 dB sensation level, random starting phase, 50 ms duration, best frequency (BF) tone. Neuron types illustrated are chopper (Ch), onset (On), pauser (P), onset-sustained (OS), low frequency (LF), onset (On) and onset chopper (OC).

Single unit recordings were also obtained from 77 neurons in the right ICc of 23 animals. Neurons were classified according to their temporal response patterns from PSTHs recorded in response to BF tones at 20 dB and 50 dB suprathreshold (using a scheme modified from Le Beau et al. (1996), Rees et al. (1997) and Syka et al. (2000)). Examples of PSTHs for each class of neuron, recorded at 50 dB SL, are shown in Fig. 2. The scheme used in the current study is described briefly. Chopper neurons demonstrated multiple peaks in their PSTH response with a CV value of 0.35 or lower. Onset-sustained neurons had an onset rate of >250 spikes/s and an onset-steady state discharge rate ratio >3 (with a CV>0.35). Pauser neurons demonstrated a reduction in spike rate (at least 7 ms in duration) relative to steady state rate of >20% within the first 15 ms of the onset of the response. Onset neurons demonstrated an onset rate: steady state rate ratio of >10 with a steady state rate of <30 spikes/s. Sustained neurons demonstrated a steady rate greater than 30 spikes/s, with no peculiar peak or trough in the response. Low frequency neurons demonstrated phase locking to the carrier frequency and had a BF of less than 500 Hz. Neurons that could not be classified by the above criteria were deemed ‘Unusual’ and are not discussed further. Using the above criteria, the following distribution of neurons was recorded: onset (On, *n*=21), onset chopper (OC, *n*=6), onset-sustained (OS, *n*=20), chopper (Ch, *n*=13), pauser (P, *n*=15), sustained (S, *n*=1) and unusual (UN, *n*=1). As so few sustained neurons were recorded in this study, these data are omitted from further analysis.

### 2.2 Forward suppression: magnitude and time course of recovery

**Fig. 3:**
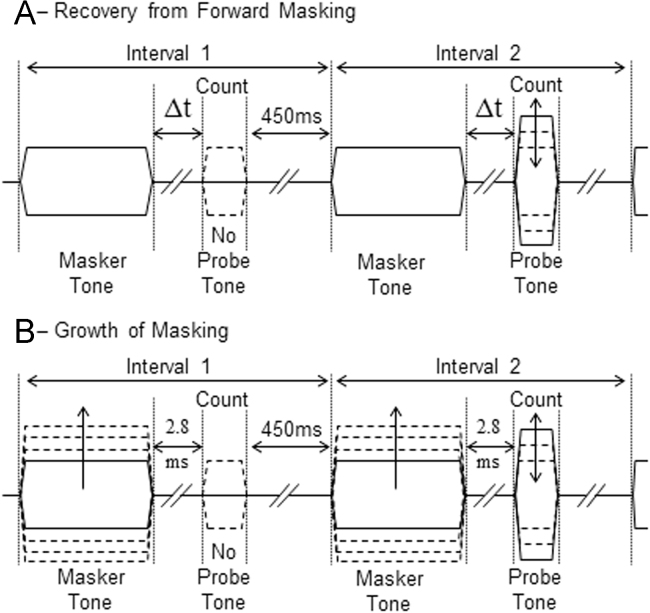
A schematic representation of the stimulus paradigm used in the PEST threshold tracking algorithm. The masker tone was always at BF and 20 dB above BF threshold. The masker tone is identical in intervals 1 and 2. The algorithm adjusts the level of the probe in interval 2 to determine the probe threshold; determined by comparison of the number of spikes evoked in a count window corresponding to the probe tone in interval 2 and an equivalently positioned count window in interval 1 (where the probe tone is absent). A. Recovery of probe threshold from forward suppression was measured using a variable masker-probe interval (Δ*t*). B. Growth of Suppression was measured by varying masker levels, with a fixed Δ*t* of 2.8 ms.

**Fig. 4:**
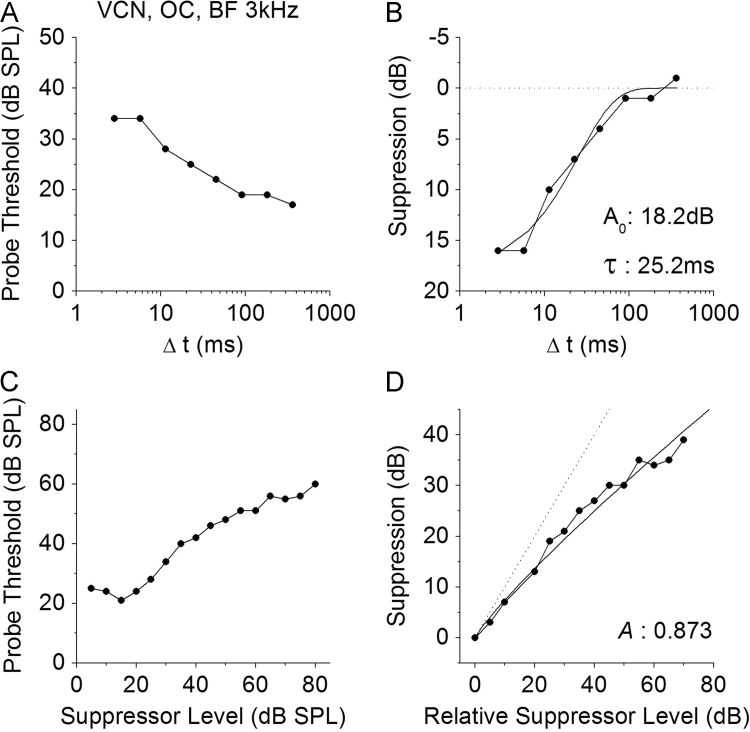
Probe threshold and forward suppression recorded in an OC unit (BF=3 kHz) from the VCN**.** A–B. Measurement of the magnitude of forward suppression as a function of Δ*t* and determination of the time course of recovery from forward suppression. A. Masked probe threshold (dB SPL) is plotted against Δ*t* (masker – probe interval, ms). B. Suppression of the probe (dB) is plotted against Δ*t*. Suppression is calculated relative to an average of the longer 2 Δ*t* threshold values. The smooth curve represents an exponential fit to the suppression data, as outlined in Methods, that was used to determine the theoretical maximum magnitude of suppression (*A*_0_), and the time constant (*τ*) used as an estimate of the recovery time course. C–D. Measurement of growth of suppression of probe threshold (with a fixed Δ*t* of 2.8 ms) as a function of masker level. C. Masked probe threshold (dB SPL) is plotted against Masker Level (dB SPL). D. Suppression of the probe (dB) is plotted against relative masker level (as described in the Methods). The smooth curve represents a power function fitting data; the exponent parameter (*A*) of the curve fit determines the slope of the growth of suppression function and gives a measure of the compression of the function as masker level increases.

For each neuron, suppressed thresholds of the probe tone were measured for Δ*t*׳s randomly varied from 2.83 ms to 362.04 ms in octave steps (Fig. 3A). Examples of suppressed probe thresholds for an OC neuron from the VCN are shown in Fig. 4A, and illustrates the trend that as Δ*t* increases suppressed threshold decreases. To quantify the amount of suppression in each neuron, the recovery curve for each neuron was normalised to an average of the suppressed threshold recorded at the 2 longest Δ*t* values, and each suppressed threshold then plotted as a magnitude of suppression (dB) relative to this average value (Fig. 4B). The ordinate is plotted in reverse order, to emphasise similarities with previous neurophysiological studies on forward suppression (e.g. Bleeck et al., 2006).

We then plotted suppression threshold as a function of Δ*t* for each unit type. Single units with suppression thresholds that fell outside the inter-quartile range for any Δ*t* were excluded from further analysis. For the remaining units the recovery data was fitted with an exponential curve (Eq. 1, see Fig. 4B), using the non-linear curve fitting routines of Origin 7.5 Pro (OriginLab Corp.), from which the time course of recovery and maximum suppression magnitude were obtained.

(1) $$y=A_{0}.e^{\left(-t/\tau \right)}$$

where

*y*=suppression (dB),
*A*_0_=suppression(dB) when Δ*t* is zero,
*t*=Δ*t* (ms),
*τ*=time constant for recovery (ms).

**Fig. 5:**
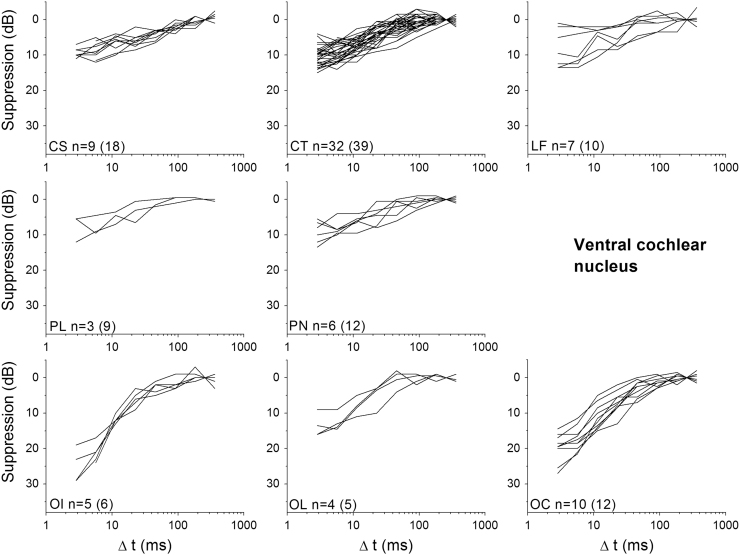
Suppression of the probe tone plotted as a function of masker – probe interval (Δ*t*) for different neuron types in the Ventral Cochlear Nucleus; sustained chopper (CS), transient chopper (CT), low frequency (LF), primary-like (PL), primary-like-with-notch (PN), onset-I (OI), onset-L (OL) and onset chopper (OC). Only those neurons producing a good exponential fit are shown here; numbers of units are indicated on each panel (along with the total number of each unit type tested, in parentheses).

**Fig. 6:**
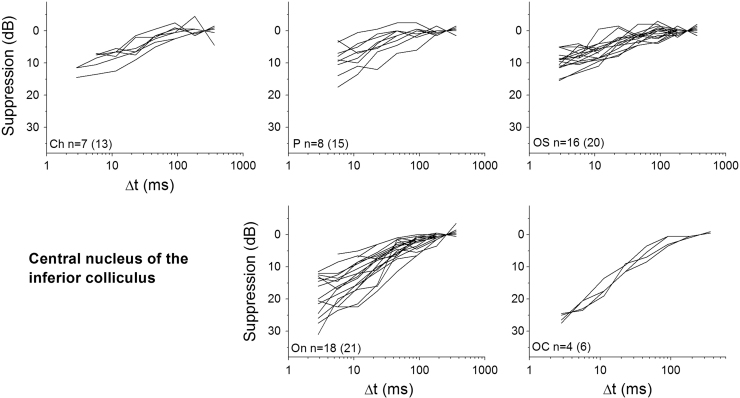
Suppression of the probe tone is plotted as a function of masker – probe interval (Δ*t*) for different neuron types in the central nucleus of the Inferior Colliculus; chopper (Ch), pauser (P), on-sustained (OS), onset (On) and onset chopper (OC). Only those non-outlier neurons producing a good exponential fit are shown here; numbers of units are indicated on each panel (along with the total number of each unit type tested, in parentheses).

**Fig. 7:**
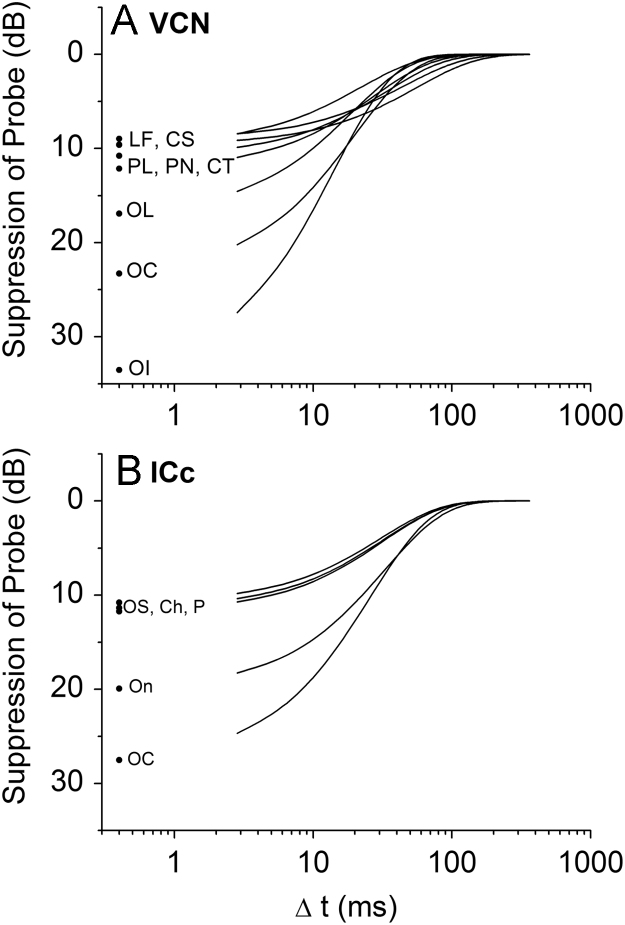
Summary recovery-from-suppression curves (calculated from the averaged parameters shown in Table 1) are plotted. Black dots near the ordinate indicate the maximal suppression magnitude (*A*_0_) for each unit type. A. Ventral Cochlear Nucleus (VCN). B. Inferior Colliculus (ICc).

Only neurons that produced a good fit to the exponential function (*R*^2^>0.7) were included in further analysis. Fig. 5, Fig. 6 show suppression as a function of Δ*t* for individual neurons of each neuron type recorded from the VCN and IC, respectively. From individual exponential fits, an average time constant (*τ*) and maximum suppression (*A*_0_) were calculated for each unit type (see Table 1). These average values were then used to calculate a summary curve for each unit type in the VCN and IC (Fig. 7A and B). In summary, for the VCN, onset units had significantly greater suppression thresholds in comparison with all other unit types (ANOVA, *F*(78,5)=21.5; *p*<0.005 with Bonferroni Post-Hoc comparison). CS units had significantly longer recovery time constants than OL/C, OI and CT units (ANOVA, *F*(78,5)=6.15; *p*<0.05 with Bonferroni Post-Hoc comparison). All other comparisons were not significant (*p*>0.05).

In the IC, onset and onset-chopper units also showed the significantly greatest magnitude of forward suppression in comparison with the other unit types. CH were significantly different from OC and ON, OC were different from OS and P, and On were different from P, (ANOVA, *F*(63,4)=11.98; *p*<0.05 with Bonferroni Post-Hoc comparison), all other comparisons were not significant. This correlates well with the degree of suppression seen in OC, OI and OL units from the VCN. However, ICc onset-type neurons produced a slower time constant for recovery from forward suppression (*τ*=32.8ms and 26.1 ms for On and OC units respectively). ICc units with a sustained discharge pattern (chopper, pauser, low-frequency and onset-sustained) produced similar forward suppression magnitudes (10.8–11.7 dB) and recovery time courses (30.6–31.6 ms) to VCN units with similar type of discharge pattern. All differences were not significant (ANOVA, *F*(63,4)=0.55; *p*>0.05).

### 2.3 Growth of forward suppression

Suppressed probe thresholds (at a fixed Δ*t* of 2.8 ms) were recorded as a function of suppressor level (Fig. 3B). Example data are illustrated for a single neuron in Fig. 4C. The growth of suppression curve for each neuron was normalised relative to the highest suppressor level where the probe threshold was at its lowest point. Suppressor level was calculated in dB above this reference point. Suppressed threshold was calculated in dB of suppression above the same reference point. These data form growth-of-suppression (GoS) functions (Fig. 4D). Visual inspection suggested these functions, like those reported by Relkin and Turner (1988) in the auditory nerve, were compressive. Therefore we fitted a simple power function to the GoS curves (Eq. 2), again using the non-linear curve fitting routines of Origin 7.5 Pro (OriginLab Corp.)

(2) $${y=x}^{A}$$

where

*y*=suppression (dB),
*x*=suppressor level (dB),
*A*=exponent

**Fig. 8:**
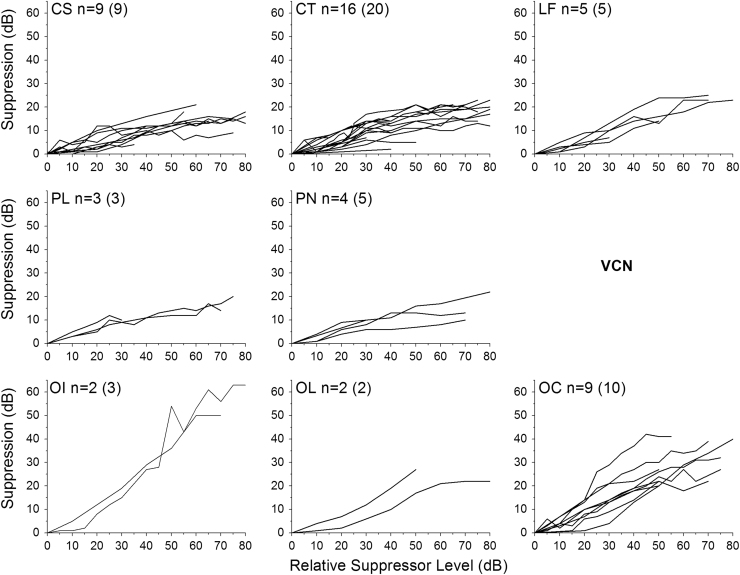
Growth of suppression functions plotted for different neuron types in the Ventral Cochlear Nucleus. Only those neurons producing a good fit to the power function are shown here; numbers of neurons are indicated on each panel (along with the total number of each neuron type tested, in parentheses).

**Fig. 9:**
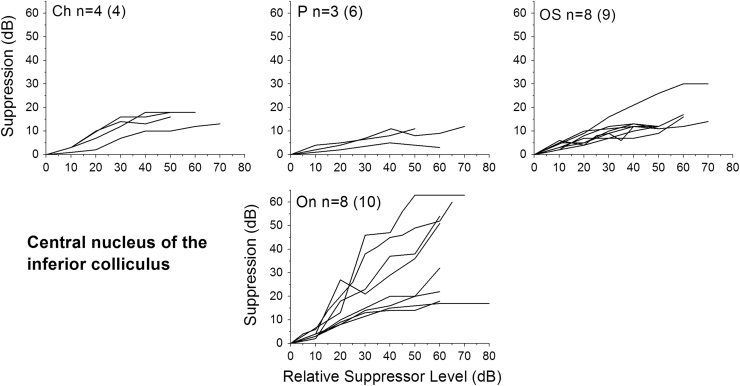
Growth of suppression functions are plotted for different neuron types in the central nucleus of the Inferior Colliculus. Only those neurons producing a good fit to the power function are shown here; numbers of neurons are indicated on each panel (along with the total number of each neuron type tested, in parentheses).

**Fig. 10:**
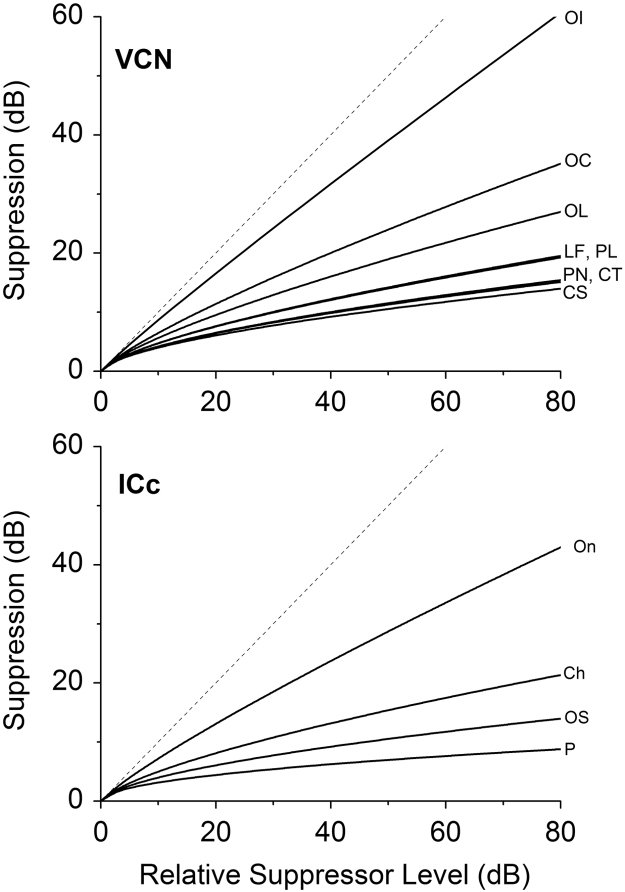
Summary growth-of-suppression curves (calculated from the averaged parameters shown in Table 1) are plotted. A. Ventral Cochlear Nucleus. B. Inferior Colliculus. Dotted line is the line of unity.

GoS functions for individual neurons were fitted, and only those curves that produced a good fit to the function (*R*^2^>0.7) were used in subsequent analyses. GoS data for individual neurons of each unit type in VCN and IC are shown in Fig. 8, Fig. 9, respectively. From these curves, it was clear that some neuron types exhibited a more compressive GoS function. The average exponent from the power fit (*A*) for each unit type (Table 1) was used to generate the summary curves, plotted in Fig. 10.

In the VCN, CS neurons were the most compressive with the lowest exponent values. CT, LF, PL and PN neurons demonstrated similar compressive functions (see Table 1). OL and OC neurons showed an intermediate degree of compression, whereas OI neurons demonstrated the least compressive growth of suppression function, having the highest average exponent value. We combined OI, OL and OC units into one group, onsets, to compare with the responses from other unit types. Onset units had significantly higher growth of suppression than CS and CT, no other comparisons were significant. (ANOVA, *F*(37,4)=4.7; *p*<0.05 with Bonferroni Post-Hoc comparison).

In the IC, Pauser neurons demonstrated the most compressive GoS, having the lowest exponent values. Onset-Sustained and Chopper neurons demonstrated similar, highly compressive functions, having low exponents. (see Table 1). Onset neurons demonstrated the least compressive growth of suppression function, having the highest average exponent value. ON were significantly higher than OS and P, no other comparisons were significant. (ANOVA, *F*(19,3)=9.66; *p*<0.05 with Bonferroni Post-Hoc comparison).

## 3 Discussion

These data represent the first comparison, to our knowledge, of forward suppression in single neurons in the VCN and central nucleus of the IC, measured using a modified psychophysical two- alternative forced-choice paradigm. This technique allowed us to describe forward suppression in terms of the dB threshold elevation of a probe tone as a function of suppressor – probe interval and as a function of suppressor level. Suppressed probe thresholds increased as a function of suppressor – probe interval, the extent of which was dependent on the type of neuron. Across the two nuclei, onset-type units showed the greatest magnitude of forward suppression and the steepest growth of suppression. Units with a sustained discharge rate to pure tone stimuli produced a lower magnitude of forward suppression, and more compressive growth of suppression. Onset units in the VCN recovered from forward suppression with a faster time course that other VCN unit types. However, onset units recorded from the IC recovered with a similar, longer, time course to other IC response types.

Recovery of suppressed probe threshold with increasing time following a suppressor tone was well described by a single exponential function, in keeping with the original study of recovery from adaptation in AN fibres (Harris and Dallos, 1979), as well as other studies in higher brainstem regions of the auditory pathway; CN (e.g. Bleeck et al., 2006) and IC (e.g. Arehole et al., 1987; Finlayson, 1999; Ingham and McAlpine, 2004).

**Fig. 11:**
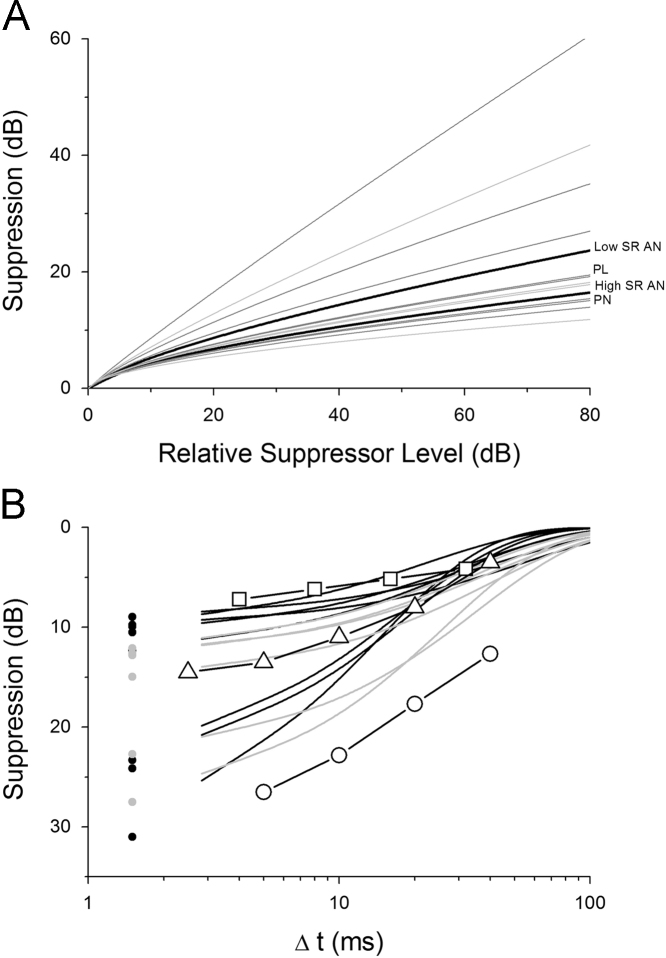
Comparison with previous studies. A. Growth of Suppression. Data derived from the published results of Relkin and Turner (1988) were analysed by the methods of the current study. The fitted power functions for high and low spontaneous rate auditory nerve fibres are plotted alongside data from the VCN and ICc (see also Table 1). B. A comparison of neural forward suppression and published psychophysical results. Black and grey lines indicate forward suppression functions from VCN and ICc neurons, respectively. Open squares represent psychophysical data, averaged across frequency, as predicted from the equations published by Jesteadt et al (1982). Open triangles represent psychophysical data published by Widin and Viemeister (1979). Open circles represent psychophysical data published by Carlyon (1988), using fixed level noise maskers.

Several studies in the AN have developed and utilised a parameter estimation by sequential tracking algorithm (PEST) to measure thresholds under forward suppression conditions (eg. Relkin and Pelli, 1987; Relkin and Turner, 1988; Relkin and Doucet, 1991). However, to our knowledge the current study is the first to use PEST techniques to quantify forward suppression in more central centres of the auditory pathway. Relkin and Pelli (1987) described a modified PEST (Taylor and Creelman, 1967) routine that allowed a rapid estimate of neural threshold (approximately 1 min) with a high degree of accuracy. The technique was then employed to measure growth of suppression in AN fibres (Relkin and Turner, 1988). High spontaneous rate fibres had reduced suppression compared to low spontaneous rate fibres. The authors noted that these suppressed auditory nerve threshold elevations were not sufficient to account for the shifts seen behaviourally, and suggested that the discrepancy could be due to “suboptimal processing … at a location central to the auditory nerve”. The growth of suppression curves for two auditory nerve fibres (Relkin and Turner, 1988, Fig. 5), bear a close resemblance to the ones shown here (see Fig. 11A). Although these authors only fitted linear functions to portions of their growth of suppression curves, it is clear that these curves show compressive features at higher masker levels in a very similar way to the CN and IC data presented here. Relkin and Turner (1988), however, also demonstrated that in 33% of AN fibres the suppressed probe threshold curve closely followed the rate-level function with increasing masker level, in some cases saturating together, in the remaining 66% of cases, threshold would continue to increase when spike rate had saturated. The reason for this difference is unclear.

A difference in the rate of recovery from forward suppression was also found between high and low spontaneous rate AN fibres. High spontaneous rate fibres (>40 spikes/s) recovered faster, whereas low spontaneous rate fibres (<20 spikes/s) recovered with a slower time course (Relkin and Doucet, 1991). Data presented here from the VCN lend support to this finding. Comparing PL and PN neurons (likely to be reflective of Relkin and Doucet׳s data), we found that low spontaneous rate neurons recovered from forward suppression with a mean *τ* of 32 ms compared to a mean *τ* of only 15 ms in high spontaneous rate units. The recovery functions of Relkin and Doucet (1991), recorded from 2–125 ms, were described as linear with log time. This is similar to the AN study of Harris and Dallos (1979), see below.

If the amount of suppression measured in the auditory nerve is insufficient to account for perceptual masking then it is logical to examine the nature of physiological suppression at higher levels of the auditory pathway. A model (Meddis and O’Mard, 2005) has suggested that coincidence detection, presumably at the level of the cochlear nucleus, could enhance the amount of suppression. To test this idea we have used the same algorithm as used for studying forward suppression in the auditory nerve by recording the responses of single units in the ventral cochlear nucleus. We have also examined the responses of single units in the central nucleus of the IC to the same stimuli. The inferior colliculus is of particular interest as it has been claimed that perceptual masking can be explained by the responses of a population of single units at this level in the marmoset (Nelson et al., 2009) where the default parameters were a 200 ms masker at BF, a SL of 40 dB and a delay between masker and probe of 10 ms. Unfortunately, because of the different parameters used, it is not clear how the measurement of threshold in marmosets compares with that used in this study and that of Relkin and colleagues. As with the findings in this study, in marmosets, units classified as onset were characterised by the greatest amount of forward suppression. In contrast to our study (and that in the auditory nerve) where the growth of suppression was well described with a compressive power-law function the growth of suppression functions were fitted with a linear function and the slope measured. While individual units could clearly show a large growth of suppression with a slope in growth of 0.5 dB/dB and hence, very close to the perceptual data, the pooled data from the marmoset IC were less convincing. The growth of suppression grew at approximately 0.5 dB /dB over the first 30 dB but thereafter grew at a more modest 0.25 dB/dB. On the whole there is a greater growth of suppression with increasing masker level for a population of units in the marmoset ICc than the guinea pig IC. There are, however, several differences between the results in the marmoset ICc and those obtained in the guinea pig (a) the proportion of type I units in the guinea pig is much smaller than that in the marmoset. This may be partly explained by the higher number of low-frequency cells in our data in comparison with that of Nelson et al. (2009). (b) The methods used to estimate threshold are not the same and may result in differences and (c) the marmoset was awake whereas the guinea pig was anaesthetised.

Whatever the reason(s) it appears that onset type units may closely follow the perceptual data while the population of units is insufficient. This leads to a common issue in sensory physiology-is perception the result of pooling information across neurons or simply the result of listening to the most sensitive neurons. While the majority of units at the level of the ICc fail to fully account for perceptual masking data the converse problem appears to exist in the auditory cortex. Recording from single and multi-units in the primary auditory cortex of the anaesthetised guinea pig Alves-Pinto et al. (2010) have shown that there is more than sufficient information in the responses of single units to explain perceptual masking (~0.8 dB/dB) and that the information would have to be sub-optimally combined to explain the human psychophysics. Of course all of these studies are ultimately handicapped by not knowing how well the marmoset or guinea pig respond to masked sounds.

The idea that enhancement of suppression begins in the IC must be considered with caution as onset units in the VCN could also demonstrate wide-dynamic range forward suppression, with no significant difference between the onset units of the IC and the VCN. In addition the responses of single units in the DCN, itself a major input to the IC have yet to be studied, along with the numerous nuclei in the SOC (e.g. Schofield and Cant, 1997).

### 3.1 Comparisons with previous physiological studies of recovery from forward suppression

As detailed in the introduction, there have been several studies of forward suppression in units in the CN. However, the novel aspect to the current study is the use of a threshold tracking algorithm to estimate suppressed probe thresholds (in dB). Previous studies have used measures based around ratios of spike counts to assess neural coding of forward suppression stimuli. In some early studies of forward suppression in the chinchilla CN, the recovery of the response to a probe tone following presentation of a suppressor was often described as linear in log-time (eg. Boettcher et al., 1990). These results were similar to those obtained in the guinea pig CN by Shore (1995). These data were recorded down to Δ*t*׳s of 5 ms. In the AN, Harris and Dallos (1979) indicated that for Δ*t*׳s of 10 ms or greater, recovery from suppression could also be described as linear in log-time. However, if shorter Δ*t*׳s were included, then AN recovery functions were better described by an exponential function. In a recent study, Bleeck et al. (2006) described the exponential time course of guinea pig CN neurons recovery from a forward suppression stimulus with Δ*t*׳s as short as 1 ms. In the current study, using Δ*t*׳s of 2.8 ms and longer, the recovery functions recorded were also well characterised (in the majority of units) by single exponential functions. These differences in the determination of recovery time course make even a qualitative comparison between studies (eg Harris and Dallos, 1979, Boettcher et al., 1990, Shore, 1995) rather difficult. As such we restrict our comparisons of CN forward suppression to the current data obtained using PEST, and recent data obtained by Bleeck et al. (2006) using PSTH measurements with short Δ*t* intervals. There is a good correspondence between the parameters associated with forward suppression recorded using either the PEST method or the PSTH spike count method of Bleeck et al. (2006) within the same neuron types. Comparing our population of PEST results with the population results of Bleeck et al. (2006), many similarities are also observed. CS neurons show limited magnitude of suppression and recover with a long time constant in both studies. The hierarchy of forward suppression magnitude for PL, PN and CT neurons is maintained in both studies. These neuron types also show similar recovery time constants in both studies. Furthermore, onset unit types show the maximal magnitude of suppression and fastest recovery time constants in both studies. However, one discrepancy is apparent in that Bleeck et al. (2006) report a greater degree of forward suppression in OC neurons, whereas the current PEST study indicates that OI neurons produce the highest suppression magnitude. Despite this, the two studies both indicate that ON neurons (OI and OL) recover from forward suppression with a faster time course than do OC neurons. Thus there are consistent and closely corresponding results for forward suppression recorded in the CN using differing methodologies. In the IC, onset neurons also show the most pronounced suppression, compared to other neuron types with more sustained firing patterns. However, IC onset neurons do not recover from forward suppression with the same rapid time course as do onset types in the CN.

There has been relatively little data published on growth-of-suppression in the CN and IC. A visual examination of the data of Boettcher et al. (1990) indicated that growth of suppression in their population of CN units was also compressive. The data for PL, PN and Chopper units indicates that GoS functions would be more compressive than that for Onset units.

### 3.2 Relationship to psychophysical findings on forward masking

Not withstanding the obvious species differences, several characteristics of forward masking have been reported from psychophysical measurements (eg. Jesteadt et al., 1982, Oxenham and Plack, 2000) that show similarities to the physiological forward suppression experiments described here. First, a greater amount of forward masking was observed when the interval between the masker and probe was short. The relationship between logarithmic scale of the interval and the amount of the masking was almost linear (cf earlier discussion). The masking effect decayed to zero regardless of the masker levels and the initial amount of masking when the Δ*t* interval reached 100–200 ms (Oxenham and Plack, 2000). Second, the increment of the threshold shift was not equal to the increment of the masker level; growth-of-masking functions had a slope <1. This slope increased at shorter Δ*t*׳s. The influence of suppressor duration on the masking effect varied across studies. There was an agreement that an increase in suppressor duration increased the masking effect when the masker was <50 ms. Fastl (1976) showed little effect on masked threshold of increasing the masker duration above 50 ms. However, other studies reported that increasing masker duration up to 200 ms had an effect on probe threshold (Kidd and Feth, 1982, Zwicker, 1984).

There appears to be a discrepancy in the amount of masking between psychophysical observations and physiological forward suppression at the level of the AN (Harris and Dallos, 1979, Relkin and Turner, 1988), and CN and IC (current study). Physiological results from anaesthetised animals and data from human behavioural studies may not be directly comparable, however, the forward masking effect in behavioural observation in the chinchilla was also greater than that of physiological observations from the anaesthetised chinchilla AN (Halpern and Dallos, 1986, Relkin and Turner, 1988). With this in mind, it would be useful to obtain behavioural forward masking data from the guinea pig (beyond the scope of the current study).

We compare physiological results from the CN and IC to the psychophysical measurements published by Widin and Viemeister (1979), Jesteadt et al. (1982) and Carlyon (1988) in Fig. 11B, at a masker sensation level of 20 dB. Jesteadt et al. (1982) published an equation to predict their forward masking data as a function of masker level. We used this equation to predict psychophysical forward masking at 20 dB masker sensation level and then averaged these predictions across frequency (Fig. 11B, open squares). Widin and Viemeister (1979) published temporal recovery data from forward masking in 2 subjects. Here, we plot an average of their subject data, normalised for masker level to correlate with the plotting conventions used in this current study (Fig. 11B, open triangles). For comparison, the data of Carlyon (1988) are plotted in Fig. 11B (open circles), although these experiments were performed using a broadband masker stimulus in contrast to the current study and the data of Jesteadt et al. (1982) and Widin and Viemeister (1979). Comparison of the predicted psychophysical forward masking data from Jesteadt et al., (1982) and the physiological forward masking data from the CN and IC (current study) produces some interesting observations. At this low relative masker level (20 dB), the physiological data demonstrate that neurons in the CN and IC are subject to a consistently greater magnitude of forward suppression, compared to predictions from Jesteadt et al. (1982). However, the data of Widin and Viemiester (1979) and Carlyon (1988) indicate that most unit types in the CN and IC (those with sustained discharge patterns) are subject to a reduced effect of forward suppression than is seen behaviourally. The psychophysical observations of Widin and Viemiester (1979) are reflected best by the responses of onset-type units in the CN and IC.

However, this does not mean that onset units play a greater role in the forward masking effect. Onset chopper neurons are widely believed to be inhibitory interneurons that do not ascend the system. They play a role in processing wideband signals with the CN to shape the responses of other neuron types to such stimuli. The only known projection of OC neurons beyond the ipsilateral CN is to the contralateral CN (e.g. Arnott et al., 2004) and therefore their influence in forward suppression is, at best, indirect. OI neurons, along with all of the sustained-discharge type CN neurons, are thought to project to higher nuclei, and therefore may help to shape the responses of higher neurons however, it is unknown whether the larger forward suppression seen in onset units determines behavioural forward masked thresholds.

### 3.3 Functional implications

The current experiments were performed in the anaesthetised guinea pig. Under such conditions, cortical influences over lower nuclei, although still present, may be less prominent (Palmer et al., 2007). As such, the potential role of attention – a cortical influence over neural processing – cannot be systematically investigated, and descending attentional modification of responses to forward suppression stimuli is likely to be important in shaping neural coding of such effects.

From such observations, it is clear that a forward suppression of evoked spikes and recovery from this suppression, will play an extensive role in auditory processing. The extent of the suppressive effect, and hence its effects of neural coding, is likely to undergo transformations above the level of the IC. A detailed knowledge of the extent of forward suppression at all levels of the system (up to the auditory cortex) is needed and should form the basis for future study to advance our understanding of this important phenomenon and how it will influence neural coding of important aspects of sound, such as pitch, speech and location. The studies should preferably use the same stimulus paradigms and threshold measuring techniques to facilitate comparison both between physiological studies and perceptual results.

## 4 Experimental procedures

Experiments were performed on guinea pigs of either sex (cavia porcellus, 310–620 g) under the guidelines of the UK Animals and Scientific Procedures Act, 1986 and the terms and conditions of a UK Home Office Project Licence granted to the fourth author (IMW). Animals were anaesthetised using urethane (1.0 g/kg, i.p. of a 25% solution, Sigma-Aldrich, UK). Additional analgesia was provided by 1 mg/kg Hypnorm (Vetpharma, UK) i.m. Surgical anaesthesia was maintained at a depth sufficient to abolish the front paw pedal withdrawal reflex. Additional doses of Hypnorm (1 mg/kg) or urethane (0.5 g/kg) were administered as required. Many of the techniques used in this study have been described in detail previously (e.g. Neuert et al., 2004; Ingham et al., 2006a).

### 4.1 Surgical procedures

All surgical and recording procedures were performed inside a sound-attenuated chamber (Industrial Acoustics Company, Winchester, UK). Incisions were pre-infiltrated with 2% lignocaine (company here, where, UK). The trachea was cannulated and in some cases, the animal ventilated artificially. Body core temperature was monitored using a rectal probe and thermostatically maintained at 37 °C using a heating blanket (Harvard Apparatus, Edenbridge, UK). The animal was placed in a stereotaxic frame and held in modified ear bars (hollow speculae) designed for the guinea pig ear.

Following a midsagittal scalp incision, the periosteum and muscles overlying the temporal and occipital bones were removed. An insulated silver wire electrode was placed on the round window of the left cochlear through a fenestration in the bulla, sealed in with petroleum jelly and used to monitor compound action potentials (CAP). The CAP thresholds recorded, across a range of frequencies, were monitored throughout the experiment. If thresholds deteriorated by >10 dB and were not recoverable (e.g. by removal of fluid from the bulla), the experiment was terminated (using an overdose of sodium pentobarbitone; Euthetal, May & Baker, Dagenham, UK).

For cochlear nucleus experiments, a craniotomy was performed to expose the left cerebellum and the overlying dura was carefully removed. The cerebellum was partially aspirated to allow direct visual placement of electrodes in the cochlear nucleus. The cavity and craniotomy were filled and sealed using 1.5% agar (Sigma-Aldrich, UK) in saline to prevent dessication of the brain. For inferior colliculus experiments, a small craniotomy exposed discrete areas of the right occipital cortex. Overlying dura was carefully removed and electrodes were placed using stereotaxic positioning into the central nucleus of the inferior colliculus. The craniotomy was sealed using 1.5% agar in saline.

### 4.2 Electrophysiological recordings

Extracellular recordings were made using glass-coated tungsten microelectrodes (Merrill and Ainsworth, 1972) mounted on a hydraulic microdrive (650 W; David Kopf Instruments, Tujunga, CA). For placement into the CN, electrodes were advanced in the sagittal plane at an angle of 45°. For placement into the IC, electrodes were advanced vertically.

Single units were isolated using broadband noise or sinusoidal tones as search stimuli. All stimuli were synthesised digitally in real time on a Dell PWS650 Workstation equipped with a DIGI 9636 optical connection to an ADI-8 DS AD-DA converter (RME Audio Products, Germany). The AD-DA converter was used for the DA conversion of stimuli and the AD conversion of neural activity (amplified ×1000 and bandpass filtered from 300–3000 Hz) and was driven using audio streaming input output (ASIO) and a software developer kit from Steinberg (Indianapolis, IN) (Lloyd, 2002). All input and output signals used a 96 kHz sampling rate per channel.

After DA conversion, stimuli were equalized (Phonic PEQ3600 graphic equaliser) to compensate for the frequency response of the speaker and ear-speculum coupler (approximately±5 dB from 0–16 kHz). Stimuli were then amplified (Rotel RB971), attenuated (programmable in 5 dB steps from 0–75 dB) and presented over a speaker (Radioshack 40–1377 tweeter modified by Mike Ravicz, MIT, Cambridge, MA) mounted into the specula in the left ear. Stimuli were monitored using a condenser microphone (type 4134 or type 4182, Bruel & Kjaer, Naerum, Denmark) attached to a calibrated 1 mm probe tube inserted into the speculum in order to sit close to the eardrum.

After AD conversion neural spikes were discriminated in software and stored on the personal computer for off-line analysis. Following isolation of a single unit, its best frequency (BF) and threshold were estimated using audiovisual criteria. Spontaneous activity was measured over a 10 s period in the absence of controlled stimulation.

### 4.3 Stimulus paradigms

To determine the classification of each unit, peri-stimulus time histograms (PSTHs) were constructed from spike times recorded in response to 250 sweeps of 50 ms BF tone bursts presented at 20 dB and 50 dB above BF threshold. Classification schemes used for CN and IC neurons are described in Section 2.

Forward Suppression was assessed by a modified version of a PEST (Parameter Estimation by Sequential Tracking) algorithm, used previously in the measurement of forward suppression in the auditory nerve (Relkin and Pelli, 1987, Relkin and Turner, 1988) that was adapted by Relkin and Pelli (1987) from the original PEST procedure developed by Taylor and Creelman (1967). This technique represents a physiological equivalent of a two-interval forced choice paradigm widely used in psychophysical experiments on the auditory system. Here, we use the PEST technique to measure masked thresholds of a probe tone preceded by a suppressor tone by a variable time period (Δ*t*).

The stimulus paradigm is illustrated in Fig. 3. The stimulus consists of 2 intervals, each beginning with the presentation of a BF suppressor tone 20 dB above threshold. The suppressor is followed by a variable silent period (Δ*t*). In interval 2 only, a probe tone was presented at Δ*t* ms following the end of the suppressor tone. Each interval was 900 ms long, to allow the presentation of the both suppressor and probe tones, with a Δ*t* interval between them and a final silent period of at least 450 ms. The duty cycle of stimulus presentation was 1900 ms. Neural spikes were counted online during stimulus presentation. The number of spikes recorded during a count window in interval 2 (adjusted for unit latency), encompassing the probe tone in interval 2, were compared with the number of spikes recorded in an equivalent count window in interval 1 (where no probe tone was presented).

The first run for a particular value of Δ*t* started with a probe tone of approximately 35 dB SPL. The algorithm increased or decreased the probe level based on results from 4 trials and the sum of a decision state. If more spikes were recorded during the second interval (with probe) than in the first interval (no probe), the decision state was increased by 1. When the number of spikes in each interval was equal the decision state was increased by 0.5. If more spikes were present in the first interval than the second, the decision state was left unchanged. After 4 trials, if the decision state value was greater than or equal to 3, the probe tone level was decreased. Otherwise, the probe level was increased. From an initial step size of 16 dB, the probe tone level step was halved in size for every reversal of step direction, so as to converge on threshold. Once the stopping step size of 1 dB is reached, the tracker algorithm continues at this step size until three more reversal points are found. The suppressed probe threshold was then recorded as the mean of the last three runs. This criterion produces threshold measurements for a 61% probability of correct detection (Relkin and Pelli, 1987).

To assess the time course of recovery from forward suppression we have measured the magnitude of forward suppression as a function of Δ*t* (see Fig. 3A). This was performed using different duration masker and probe tones. Initially stimulus parameters were chosen to correspond to previous studies in this laboratory on forward suppression (Bleeck et al., 2006). Suppressors and probes consisting of equal duration (50 ms) BF tones presented 20 dB above the audio-visually determined threshold for each neuron. Δ*t* was varied from 2.83 ms to 362.04 ms in octave steps. However, this particular configuration produced little suppression of the probe tone. Thus, for the experiments presented here, a stimulus configuration of 100 ms suppressor, 25 ms probe was used (similar to Relkin and Pelli, 1987). The magnitude of forward suppression was also investigated as a function of suppressor level (with a fixed Δ*t* of 2.83 ms), using the 100 ms masker 25 ms probe configuration (Fig. 3B).

## Figures and Tables

**Table 1:** Forward Suppression Parameters in the VCN and IC. Listed beside each class of neuron are (i) the number of units recorded, (ii) the number of these units which did not produce a good exponential fit of suppressed threshold vs. Δ*t*, (iii) the number of neurons classified as outliers by a boxplot analysis, and (iv) the number of neurons included in the average. Values are given as mean±standard deviation. One way Analysis of Variance with multiple comparisons was used to test for significant differences in parameter values between different neurons. The following statistical differences were observed: (a) For comparison purposes the groups OL and OC were grouped together. The magnitude of suppression was significantly greater for both OI and OL/C groups when compared with the other VCN unit types (*p*<0.05, ANOVA, Bonferroni׳s Method for multiple comparisons); (b) Units classified as CS had a significantly longer time constant when compared with OI, OL/C and CT units (*p*<0.05, ANOVA, Bonferroni׳s Method for multiple comparisons) (c) We grouped together OI, OL and OC units in the VCN to form a single onset group to compare the growth of suppression. The onset group showed significantly stronger growth of suppression when compared with chopper units (*p*<0.05, ANOVA, Bonferroni׳s Method for multiple comparisons) but not when compared with PL or PN units. (d) In the ICc both OC and On units showed significantly greater suppression than other ICc unit types (*p*<0.05, ANOVA, Bonferroni’s Method for multiple comparisons). There was no significant difference in the magnitude of suppression between onset-sustained, chopper and pauser unit types. There was also no significant difference in the time constant of recovery between unit types in the ICc. (e) The growth of suppression was greater for onset units when compared with both onset-sustained and pauser units (*p*<0.05, ANOVA, Bonferroni׳s Method for multiple comparisons). Data derived from the auditory nerve (AN) study of Relkin and Turner (1988) are included for comparison. (n/a; data not available).

Unit type	Maximum suppression magnitude (*A*_0_), dB	Time constant (*τ*), ms	Growth of suppression, power exponent, *A*
**Ventral cochlear nucleus**			
**CS** (18, 4, 5, 9)	9.6±2.3	55.6±23.2^b^	0.601±0.117
**CT** (39, 6, 1, 32)	12.1±2.9	27.2±17.7	0.619±0.160
**LF** (10, 3, 0, 7)	8.9±5.6	45.8±32.8	0.678±0.088
**PL** (9, 4, 2, 3)	9.6±3.5	27.2±12.8	0.675±0.043
**PN** (12, 4, 2, 6)	10.8±3.2	33.6±23.8	0.624±0.081
**OI** (6, 0, 1, 5)	33.5±9.1**^a^**	14.1±6.0	0.937±0.015^**c**^
**OL** (5, 0, 1, 4)	16.9±3.8 ^**a**^	18.8±10.7	0.752±0.071^**c**^
**OC** (12, 0, 2, 10)	23.3±4.2^**a**^	19.9±8.6	0.812±0.075^**c**^

**Inferior colliculus**			
**Ch** (13, 4, 2, 7)	11.7±2.4	31.5±12.9	0.699±0.144
**P** (15, 4, 3, 8)	11.3±4.1	31.6±29.2	0.496±0.142
**OS** (20, 1, 3, 16)	10.8±3.2	30.6±18.8	0.659±0.068
**On** (21, 0, 3, 18)	19.9±7.1^**d**^	32.8±214.0	0.858±0.136^**e**^
**OC** (6, 0, 2, 4)	28.0±1.8^**d**^	26.1±7.0	n/a

**Auditory nerve**			
Low SR	n/a	n/a	0.722±0.015
High SR	n/a	n/a	0.639±0.011
